# Factors Relating to Late Presentation of Patients With Breast Cancer in Area 2 KwaZulu-Natal, South Africa

**DOI:** 10.1200/JGO.2016.008060

**Published:** 2017-03-08

**Authors:** Sharon R. Čačala, José Gilart

**Affiliations:** All authors: Grey’s Hospital, University of KwaZulu-Natal, Pietermaritzburg, South Africa

## Abstract

**Purpose:**

Patients with breast cancer (BC) in Area 2 KwaZulu-Natal, South Africa, often present with advanced disease. We performed a review of the patients’ sociodemographic characteristics and their reasons for late presentation to identify what changes could be made to improve time to presentation.

**Patients and Methods:**

Fifty women with T1, T2, T3, or T4 BC were assessed for sociodemographic data. Patients in T3 and T4 groups were asked to provide reasons for late presentation.

**Results:**

Of 172 patients, 50 had T2, T3, or T4 BC, and 22 had T1. Age ranged from 23 to 100 years (average, 56 years). There was no significant difference in age for different tumor sizes. The average size of a T1 tumor was 1.8 cm; T2, 3.6 cm; T3, 11.4 cm; and T4, 14.8 cm. Regarding education, 19% of patients had never attended school (T1, 5%; T2, 12%; T3, 22%; T4, 32%), and 19% had completed their education (finished 12th grade). The average education level was 6th grade. Patients with larger tumors had less education (*P* < .05). Of the patients who lived in rural areas, 41% had T1, 52% had T2, 66% had T3, and 78% had T4 tumors (*P* < .01). Patients with larger tumors were associated with having less electricity in their homes than patients with smaller tumors (*P* < .05). Patients presented with a variety of symptoms. A breast lump was the presenting complaint in 96% of T1 and T2, 68% of T3 and 32% of T4; with a nipple or skin change, 2% of T3 and 8% of T4; because their families insisted, 6% of T3 and 8% of T4; because of pain, 24% of T3; and because of pain with malodorous smell, 50% of T4. Patients’ reasons for late presentation were fear (40%), not aware of disease severity (40%), fear of losing a breast (40%), referral problems (34%), financial problems (8%), and transportation problems (6%). Approximately 33% sought medical help from traditional healers, and 65% regularly attended clinics.

**Conclusion:**

Patients who presented late often lived in rural areas with fewer amenities (such as having no electricity in their homes), less education, and poor understanding of BC. Pictorial information about BC needs to be introduced to people who live in rural communities, and opportunistic screening needs to be provided at local clinics.

## INTRODUCTION

Area 2 in KwaZulu-Natal, South Africa, has a population of around 3.5 million people; 75% are younger than age 35 years. The population is largely rural and the majority are low income.^1^ Approximately 300 new cases of breast cancer (BC) are diagnosed in the public sector each year. From previous local studies, 48% of newly diagnosed patients with BC (54% of black women) required neoadjuvant or palliative oncologic treatment of inoperable or metastatic disease because of late presentation.^2^ These were women with American Joint Committee on Cancer (AJCC) classification of T4 or N3 or M1 BC.^3^ This could be attributed in part to sociodemographic factors. This study was undertaken to review the validity of this assumption and to identify where changes could be made to promote earlier presentation of women with BC.

## PATIENTS AND METHODS

The AJCC staging system provides a strategy for grouping patients with respect to prognosis.^3^ TNM staging assesses the tumor (T), regional nodes (N), and distant metastases (M). Using the T component of this classification for BC, this prospective study (undertaken at Grey’s Hospital, KwaZulu-Natal, Pietermaritzburg, South Africa, during 2014) aimed to recruit 50 patients in each of the four T stages of BC. This was an arbitrary number that was considered obtainable within 1 year in our hospital. Sociodemographic data, presenting complaint of the patient, health-seeking behaviors (clinic visits), and information on tumor size were collected. Those with T3 or T4 BC were asked why they delayed presenting and why they presented now. The information was entered into a local BC database approved by an ethics committee (Biomedical Research Ethics Committee Reference Number: BCA434/14). Fisher’s exact test was used to compare categorical variables. All tests were two-tailed and significance was set at *P* < .05. The duration of symptoms was not evaluated because we found that patients’ responses to this question were often unreliable and were not concordant with the clinical picture. Women with T3 or T4 BC often gave the duration of symptoms as a few weeks, which did not fit the clinical signs.

## RESULTS

**Fig 1:**
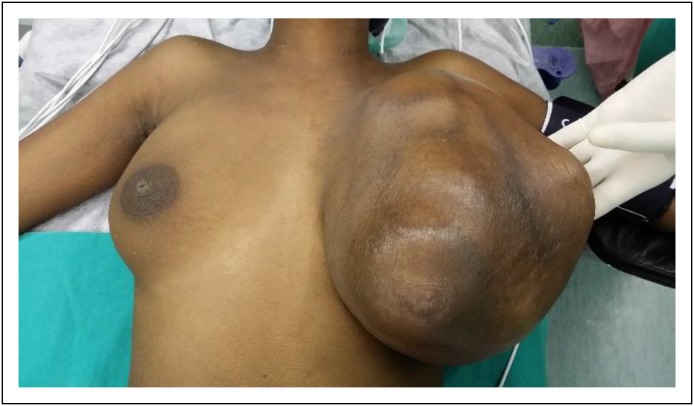
Left T3 breast cancer.

**Fig 2:**
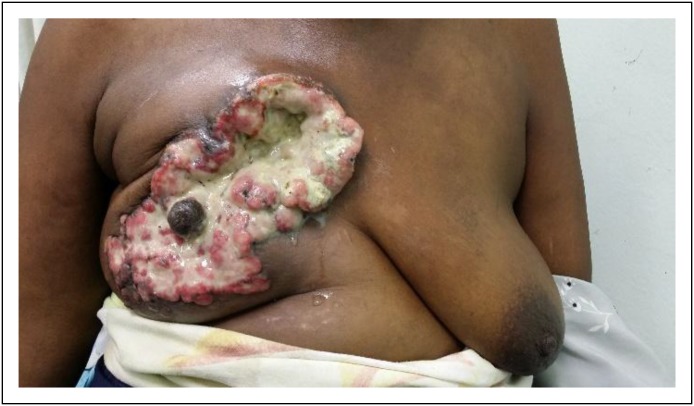
Right T4c breast cancer.

During 2014, data were collected prospectively on 50 women with BC in each T2, T3, and T4 group, but only 22 women with T1 BC were diagnosed and reviewed. Overall, 172 patients with BC were evaluated. The mean age was 56 years (range, 23 to 100 years). There was no significant difference in the average ages among the tumor size groups (T1, 55 years; T2, 59 years; T3, 52 years; T4, 57 years.) Ethnicity was self-reported: black, 82%; Indian, 9%; white, 5%; and colored, 4%. The average size of the tumors was 1.4 cm in the T1 group and 3.6 cm in the T2 group. A T3 tumor is > 5 cm; in this study, the mean T3 size was 11.4 cm (Fig 1). T4 lesions (invading the chest wall, ulcerated, having peau d’orange or inflammatory BC) had associated masses with a mean size of 14.8 cm (Fig 2).

**Fig 3:**
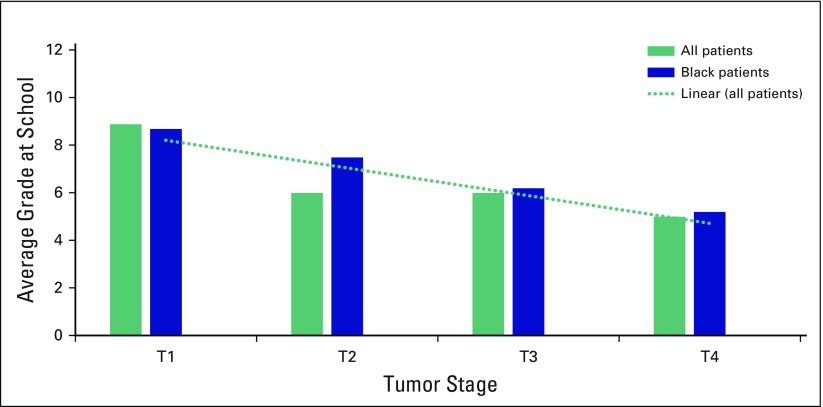
Level of education for patients in each tumor stage group.

Regarding education, 19% of patients had never attended school, and only 19% had completed high school (grade 12). The average level of education was 6th grade. Approximately one third, 32%, of patients with T4, 22% with T3, 12% with T2, and 5% with T1 lesions had never been to school. Patients with larger tumors had less education (*P* < .05). This was significant for all ethnicities and for black patients alone (Fig 3). Only 27% of patients were employed, and there was no difference with respect to tumor size and employment. Women with more advanced tumor sizes were more likely to reside in rural areas (*P* < .01) and to have no electricity in their homes (*P* < .05; Table 1).

**Table 1:**
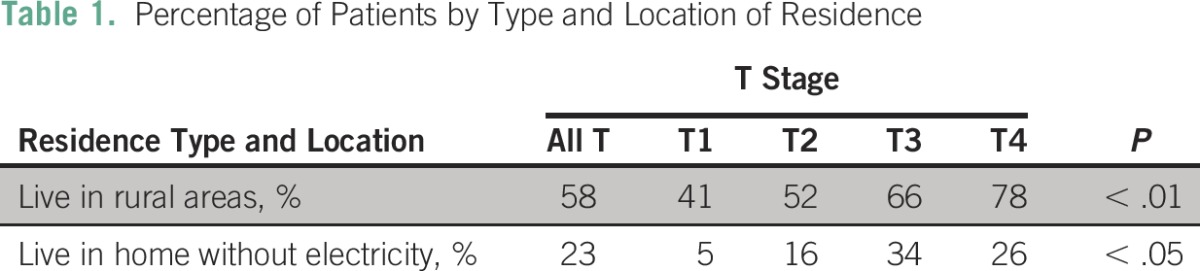
Percentage of Patients by Type and Location of Residence

Patients with T3 or T4 BC were asked why they had delayed presenting to a clinic or doctor; then they were given a list of possible reasons and asked to indicate which of the reasons applied to them. Forty percent of patients were not aware that the lump could be cancer, 40% did not understand the severity of their disease, 40% stayed away out of fear, 40% were afraid of losing a breast, and 34% had difficulty with the referral system and rural clinics. Only a few patients had financial (8%) or transportation (6%) issues. Approximately 33% of women with T3 or T4 cancers had seen a traditional healer before presenting, and one patient was a Sangoma (traditional healer) herself.

**Fig 4:**
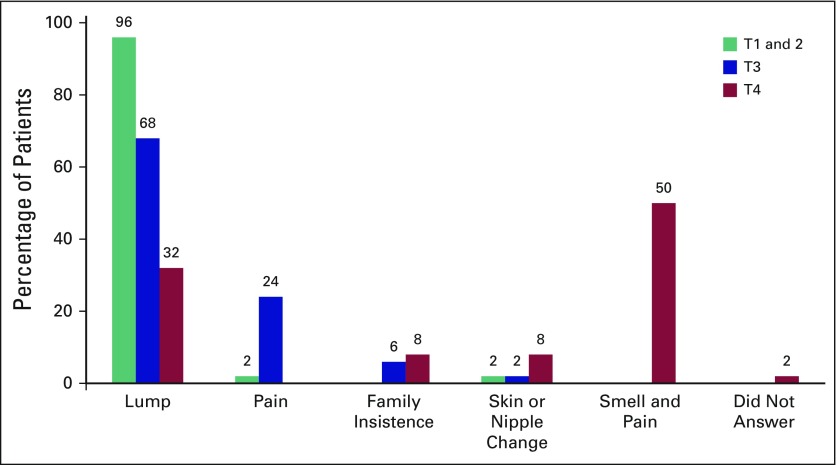
Main presenting complaint for patients in each of the four tumor stage groups.

The main presenting complaint for women with T1 or T2 tumors was a lump (96%). Nipple discharge and breast pain were the other main concerns. The majority of women with T3 lesions (68%) presented because of a breast lump, and another 24% presented because of breast pain. The main concern for patients with T4 tumors was the malodorous smell with pain (Fig 4). Local clinics were attended monthly by 65% of all the study patients, 55% attended for antihypertensive medications, and 17% attended for antiretroviral medication (of whom 7% received treatment for hypertension as well). Overall, 17% were HIV positive.

## DISCUSSION

BC is the most common cancer in women, accounting for 25.1% of all cancers. Incidence of BC in developed countries is higher, whereas relative mortality is highest in less developed countries. Educating women is suggested in all countries to help achieve early detection and treatment.^4^ In this study, we found that patients presenting with advanced cancers (T3 or T4 tumors) had lower levels of education. This may reflect their lack of understanding of the disease severity (40%) and that a breast lump might be malignant (40%). In Mongolia, employment and education were found to be associated with greater awareness of both cervical and breast cancers.^5^

This study showed that the majority of patients lived in rural areas, which was significant with respect to tumor size. Other studies showed that women in rural areas had lower levels of knowledge of BC than those in urban areas.^6^ In a review of 1,590 participants from Bangladesh, 81.9% had never heard of BC, and awareness of BC was inversely associated with rural dwelling, primary education, and having no education.^7^

The lack of electricity in women’s homes for the 30% of patients with T3 or T4 tumors may mean less exposure to media (radio and television) and less awareness of BC. Studies have shown that books, magazines, brochures, and television were among the most common sources of information regarding BC.^6^

Forty percent of the women in our study with T3 or T4 BC were fearful of the hospital, 40% were concerned about loss of a breast, and approximately 33% had seen a traditional healer. Some of the same themes were identified as causes of late presentation in a study from Ghana, namely a lack of knowledge about BC, fear of cancer treatment and its outcomes, poverty, and traditional and spiritual beliefs.^8^ A strong influence of complementary and alternative medicine was cited as a reason for delay in presentation in Malaysia.^6^

A systematic review of 18 studies (a total of 6,183 participants) of black women with BC found that delay was multifactorial, individual, and complex. Factors that contributed to delay included poor knowledge of symptoms and risk factors, fear of detecting a breast abnormality, fear of cancer treatments, fear of partner abandonment, embarrassment at disclosing symptoms to health care professionals, taboos, and the stigma of having cancer. The review stated that one of its limitations was the paucity of studies conducted outside the United States.^9^ In Africa, ignorance, the use of alternative medicine, and a fear of surgery were common reasons given for late presentation.^10^

A way to overcome these issues and improve time to presentation is through better education and awareness of BC. This should be done with visual aids such as videos that could be shown at local clinics. Discussion about BC with pictures of what to look for and who to see and how to proceed once a problem is identified would need to be included. Review could be undertaken by the community caregivers or nursing staff at the local clinics who could then give direct referrals to breast clinics in regional hospitals. It must be emphasized that early BC presentation may help preserve the breast and improve survival. Written pamphlets would be less useful in promoting BC awareness because many patients in our study could not read; 27% of patients with T3 or T4 BC had never attended school, and the average grade completed (6th grade) was less than half that required for a high school education (12th grade). Nursing staff in the local clinics should be taught to do breast examinations and to opportunistically screen women. Screening could take place when patients present for their monthly supply of prescription medication: 65% of our study patients were receiving regular medications from local clinics. These measures to improve BC education along with providing supportive community personnel and easy referral systems are means for decreasing patient and system delays.

In conclusion, patients with BC who present late are often from rural areas with few amenities. They tend to have lower levels of schooling, poor understanding of BC, and are often fearful of hospitals and surgery. Pictorial means of conveying information about BC needs to be introduced to the rural community, along with opportunistic screening at local clinics. Methods of improving BC awareness and implementing easy referral systems to breast clinics should improve time to presentation and improve survival for patients with BC.

## References

[1] Province of KwaZulu-Natal: Department of Health Strategic Plan 2010-2014. http://www.kzncomsafety.gov.za/Portals/0/Documents/Strategic%20Plans/2011%20-%202014%20Strategic%20Plan.pdf

[2] Čačala SR: Breast cancer characteristics among ethnic groups in the public sector of Area 2 KwaZulu-Natal. Presented at the Annual Scientific Meeting of the Breast Interest Group of Southern Africa, 2014

[3] American Joint Committee on Cancer: Breast Cancer Staging, Quick References, Cancer Staging Posters. https://cancerstaging.org/references-tools/quickreferences/Documents/BreastMedium.pdf

[4] Ghoncheh M, Pournamdar Z, Salehiniya H (2016). Incidence and mortality and epidemiology of breast cancer in the world. Asian Pac J Cancer Prev.

[5] Yerramilli P, Dugee O, Enkhtuya P (2015). Exploring knowledge, attitudes, and practices related to breast and cervical cancers in Mongolia: A national population-based survey. Oncologist.

[6] Khan TM, Leong JP, Ming LC (2015). Association of knowledge and cultural perceptions of Malaysian women with delay in diagnosis and treatment of breast cancer: A systematic review. Asian Pac J Cancer Prev.

[7] Islam RM, Bell RJ, Billah B (2016). Awareness of breast cancer and barriers to breast screening uptake in Bangladesh: A population based survey. Maturitas.

[8] Asoogo C, Duma SE (2015). Factors contributing to late breast cancer presentation for health care amongst women in Kumasi, Ghana. Curationis.

[9] Jones CE, Maben J, Jack RH (2014). A systematic review of barriers to early presentation and diagnosis with breast cancer among black women. BMJ Open.

[10] Ekanem VJ, Aligbe JU (2006). Histopathological types of breast cancer in Nigerian women: A 12-year review (1993-2004). Afr J Reprod Health.

